# Engineering and systems-level analysis of *Saccharomyces cerevisiae* for production of 3-hydroxypropionic acid via malonyl-CoA reductase-dependent pathway

**DOI:** 10.1186/s12934-016-0451-5

**Published:** 2016-03-15

**Authors:** Kanchana R. Kildegaard, Niels B. Jensen, Konstantin Schneider, Eik Czarnotta, Emre Özdemir, Tobias Klein, Jérôme Maury, Birgitta E. Ebert, Hanne B. Christensen, Yun Chen, Il-Kwon Kim, Markus J. Herrgård, Lars M. Blank, Jochen Forster, Jens Nielsen, Irina Borodina

**Affiliations:** The Novo Nordisk Foundation Center for Biosustainability, Technical University of Denmark, Kogle Allé 6, 2970 Hørsholm, Denmark; Institute of Applied Microbiology, RWTH Aachen University, Worringer Weg 1, 52056 Aachen, Germany; Department of Biology and Biological Engineering, Chalmers University of Technology, Kemivägen 10, 41296 Gothenburg, Sweden; The Novo Nordisk Foundation Center for Biosustainability, Chalmers University of Technology, Kemivägen 10, 41296 Gothenburg, Sweden; Evolva Biotech A/S, Lersø Park Allé 42-44, 2100 Copenhagen, Denmark; Bio R&D Center, Paikkwang Industrial Co. Ltd, 57 Oehang-4 gil, Gunsan-si, Jellabukdo Korea

**Keywords:** 3-Hydroxypropionic acid, *Saccharomyces cerevisiae*, Redox metabolism, Metabolic engineering

## Abstract

**Background:**

In the future, oil- and gas-derived polymers may be replaced with bio-based polymers, produced from renewable feedstocks using engineered cell factories. Acrylic acid and acrylic esters with an estimated world annual production of approximately 6 million tons by 2017 can be derived from 3-hydroxypropionic acid (3HP), which can be produced by microbial fermentation. For an economically viable process 3HP must be produced at high titer, rate and yield and preferably at low pH to minimize downstream processing costs.

**Results:**

Here we describe the metabolic engineering of baker’s yeast *Saccharomyces cerevisiae* for biosynthesis of 3HP via a malonyl-CoA reductase (MCR)-dependent pathway. Integration of multiple copies of *MCR* from *Chloroflexus aurantiacu*s and of phosphorylation-deficient acetyl-CoA carboxylase *ACC1* genes into the genome of yeast increased 3HP titer fivefold in comparison with single integration. Furthermore we optimized the supply of acetyl-CoA by overexpressing native pyruvate decarboxylase *PDC1*, aldehyde dehydrogenase *ALD6*, and acetyl-CoA synthase from *Salmonella entericaSEacs*^*L641P*^. Finally we engineered the cofactor specificity of the glyceraldehyde-3-phosphate dehydrogenase to increase the intracellular production of NADPH at the expense of NADH and thus improve 3HP production and reduce formation of glycerol as by-product. The final strain produced 9.8 ± 0.4 g L^−1^ 3HP with a yield of 13 % C-mol C-mol^−1^ glucose after 100 h in carbon-limited fed-batch cultivation at pH 5. The 3HP-producing strain was characterized by ^13^C metabolic flux analysis and by transcriptome analysis, which revealed some unexpected consequences of the undertaken metabolic engineering strategy, and based on this data, future metabolic engineering directions are proposed.

**Conclusions:**

In this study, *S. cerevisiae* was engineered for high-level production of 3HP by increasing the copy numbers of biosynthetic genes and improving flux towards precursors and redox cofactors. This strain represents a good platform for further optimization of 3HP production and hence an important step towards potential commercial bio-based production of 3HP.

**Electronic supplementary material:**

The online version of this article (doi:10.1186/s12934-016-0451-5) contains supplementary material, which is available to authorized users.

## Background

For more than a century, coal, petroleum and natural gas have been the primary feedstocks for the chemical industry. However, the fossil resources are diminishing whilst demand for chemicals is ever increasing. The environmental pollution associated with discarding spent chemicals and materials is also gaining focus both on political and consumer levels. To address this issue a lot of effort is going into developing novel biotechnological strategies for producing chemicals from renewable feedstocks [1, 2], such as sugar cane, starch, biomass hydrolysates, agricultural waste, etc.

One of the chemicals that have attracted particular interest is 3-hydroxypropionic acid (3HP), which can serve as a precursor for acrylic acid and its derivatives. Additionally 3HP can be polymerized alone or in compositions to obtain biodegradable polyesters. The world annual production of acrylic acid and its esters is anticipated to increase to 6 million tons by 2017 (“Acrylic Acid: 2014 World Market Outlook and Forecast up to 2018”, January 2014). Acrylates find application in a wide range of consumer products, e.g. personal care products, adhesives, coatings and paints, and the annual total market size is exceeding USD12 billion. One particularly important application of acrylic acid is for the production of superabsorbent polymers, which constitute a significant part of baby diapers and incontinence products.

3HP occurs naturally in the metabolism of some thermophilic archaea as an intermediate of 3HP-dependent carbon assimilation cycle. In these organisms, 3HP is synthesized from malonyl-CoA via combined action of malonyl-CoA reductase (MCR, EC 1.2.1.75) and 3-hydroxypropionate dehydrogenase (EC 1.1.1.298 or EC 1.1.1.59)/3-hydroxyisobutyrate dehydrogenase (EC 1.1.1.31) or via a bi-functional MCR (EC 1.2.1.75/1.1.1.298) alone. Recombinant production of 3HP in *Escherichia coli*, expressing MCR from *Chloroflexus aurantiacus*, was first reported by Cargill, Incorporated [3] and was further developed and demonstrated on a pilot scale by OPX Biotechnologies [4]. Production of 3HP by *E. coli* is, however, hampered by the requirement to produce at pH close to neutral, which results in production of dissociated form of 3HP. It is therefore advantageous to use an acid-tolerant host, such as yeast, as it is hereby possible to directly produce the acid form of 3HP. We recently described the application of a synthetic pathway towards 3HP via β-alanine intermediate in *Saccharomyces cerevisiae*, and the final strain produced 3HP at a titer of 13.7 ± 0.3 g L^−1^ with a 0.14 ± 0.0 C-mol C-mol^−1^ yield on glucose in controlled fed-batch fermentation in defined mineral medium at pH 5 [5]. Production of 3HP via MCR in baker’s yeast *S. cerevisiae* was also reported. The final strain produced up to 0.5 g L^−1^ in shake flask cultivation [6]. Our aim was to examine if it was possible to further improve the 3HP production via malonyl-CoA pathway through a rational metabolic engineering approach. In the future the malonyl-CoA and β-alanine routes towards 3HP could be combined in one strain.

Here we describe the metabolic engineering of *S. cerevisiae* for high-level production of 3HP via the MCR pathway and characterize the production strain by ^13^C flux and transcription analysis.

## Results and discussion

### Production of 3HP in *S. cerevisiae* by overexpression of acetyl-CoA carboxylase and MCR

**Fig. 1:**
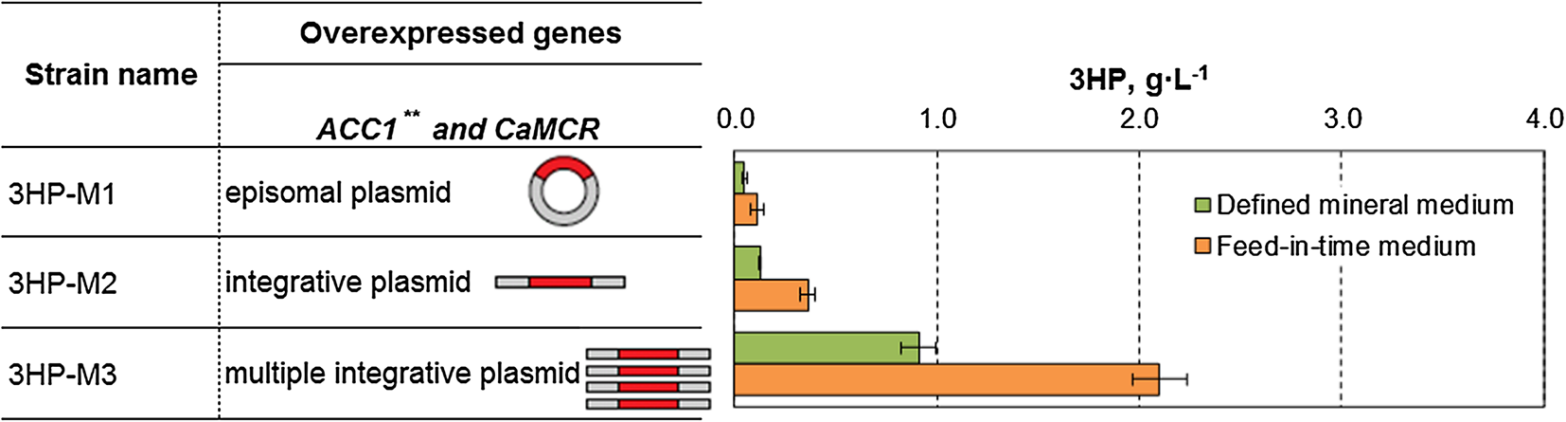
Production of 3HP by *S. cerevisiae* expressing *CaMCR* and *ACC1* ^**^ genes. The genes were overexpressed in yeast strains from a 2μ-episomal vector, a single integrative vector or a multiple integrative vector. Displayed are the average values ± standard deviations for 12 individual clones from each strain. Experiments were carried out in triplicates

For establishing 3HP production via the malonyl-CoA pathway in *S. cerevisiae*, we decided to co-express the gene encoding the bi-functional MCR from *C. aurantiacus* (*CaMCR*) together with the mutated acetyl-CoA carboxylase (Acc1^*S659A,S1157A*^). Acc1p is involved in the conversion of acetyl-CoA into malonyl-CoA, and is the rate-limiting step in fatty acid biosynthesis [7]. The double mutations in Acc1p were shown to enhance the activity and improve the production of malonyl-CoA-derived compounds, such as 3HP and fatty acid ethyl esters [8]. In this study, we over-expressed *CaMCR* and mutated *ACC1*^*S659A,S1157A*^ (ACC1^**^) genes under the control of *P*_*PGK1*_ and *P*_*TEF1*_ promoters, respectively, in *S. cerevisiae*. We investigated the effect of expression levels of *CaMCR* and ACC1^**^ on 3HP production by introducing these genes into *S. cerevisiae* via either a 2μ-based episomal plasmid, a single integration plasmid, or a multiple integration plasmid (TY4-plasmid). The TY4-plasmid is designed for integrating in multiple copies at long terminal repeats (LTRs) of retrotransposon of the TY4 family [9]. As the transformants derived from either episomal or multiple integrative plasmids are expected to have different copy numbers of the expression vector, we randomly screened a minimum of 12 clones to test for 3HP production (Fig. 1). The resulting strains were evaluated for 3HP production on defined mineral medium containing glucose as the sole carbon source (imitating a batch process), and on feed-in-time medium for *S. cerevisiae* to simulate 3HP production in a fed-batch process. The feed-in-time medium contains higher polysaccharide and an enzyme, which degrades the polysaccharide and releases glucose. The amount of the enzyme defines the rate of glucose release and was chosen here to obtain a carbon-limited cultivation. Single integration of *CaMCR* and ACC1^**^ into the genome led to improvement of 3HP titer in both defined mineral and feed-in-time media compared to 3HP titer by the strain carrying genes on an episomal plasmid. Furthermore, expression of *CaMCR* and ACC1^**^ in multiple copies via TY4-mediated integration had a further positive effect and led to a threefold improvement of 3HP titer, when compared to a *S. cerevisiae* strain bearing a single integrative vector with the same genes. However, a significant decrease in the maximum specific growth rate (μ_max_) was also observed in the strain carrying TY4-*CaMCR*–ACC1^**^ compared to the reference strain (Additional file 1: Fig. S1). We then used qPCR to analyze the copy number of *CaMCR*–ACC1^**^ genes in the strains with TY4-integration and found that the inserted genes were present at 3–4 copies, whereas only 0.5–1 copy of the inserted genes was detected in strains carrying an episomal plasmid (in addition to the inserted ACC1^**^ gene, also the native *ACC1* gene was detected, therefore the total copy number of *ACC1* was always one copy higher than of the *CaMCR*). These results show that the copy number of *CaMCR* and ACC1^**^ had a distinct effect on 3HP production and the cellular growth. The copy number of the genes below one for the episomal plasmid points to strain instability and population heterogeneity. The episomal vector has a large size of 17 kb and may be difficult to maintain in yeast.

The benefits of expressing multiple genes from integrative vectors rather than from episomal vectors has been reported earlier [10]. The strains are more stable and homogenous expression of all genes within the yeast population was observed. Furthermore, optimization of the critical enzymes in the pathway by multiple integration at TY4 elements has been reported to result in fourfold increase 3HP production via the β-alanine pathway [5]. In this study, the impaired growth in the strain carrying TY4-*CaMCR*–ACC1^**^ might be caused by the higher activity of Acc1p and MCR, which likely resulted in high metabolic burden for the cells. Similar effect of high Acc1p activity on the cellular growth has been previously reported in both yeast and bacteria [8, 11]. In addition, the higher activity of MCR could also result in competition with the fatty acid biosynthesis for the substrate, malonyl-CoA. A possible solution to this problem could be dynamic control of expression of the biosynthetic genes, where they are mostly expressed in the production phase, but not in the growth phase.

### Improvement of 3HP production by increasing the supply of acetyl-CoA precursor

**Fig. 2:**
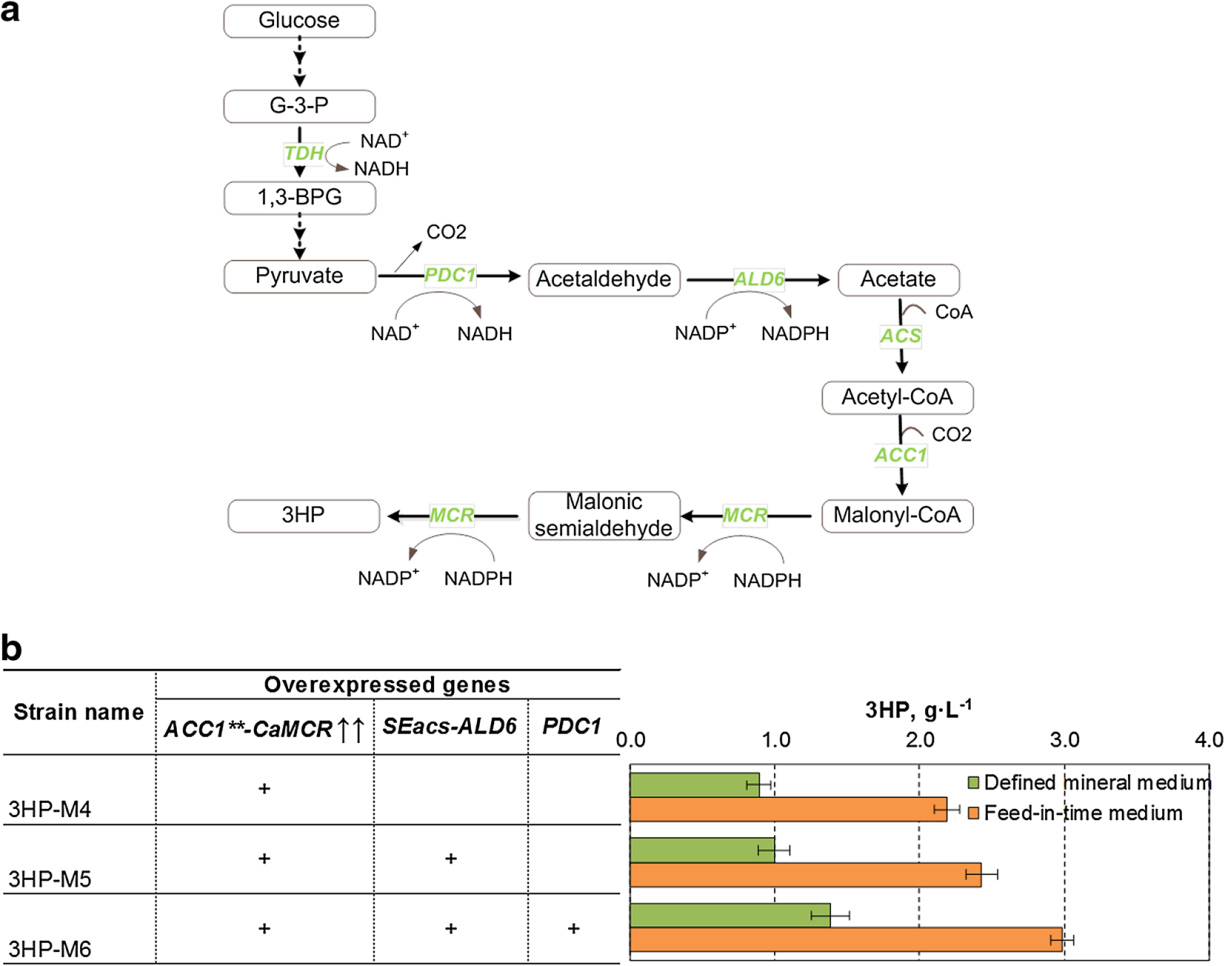
Influence of overexpression of precursor supply genes (*SEacs* ^*L641P*^, *ALD6* and/or *PDC1*). **a** Schematic pathway representing 3HP biosynthesis in *S. cerevisiae*. *G-3-P* glyceraldehyde3-phosphate, *1,3-BPG* 1,3-Bisphosphoglycerate. **b** 3HP titer in engineered strains grown in defined mineral or feed-in-time media. *Error bars* represent the standard deviations in 12 biological replicates. *G-3-P* glyceraldehyde-3-phosphate, *3HP* 3-hydroxypropionic acid

It was previously shown that overexpression of acetyl-CoA synthase derived from *Salmonella enterica* (*SEacs*^*L641P*^) and of the native aldehyde dehydrogenase (*ALD6*) enhanced the flux towards acetyl-CoA and resulted in higher isoprenoids [12] or 3HP concentration [6]. For another acetyl-CoA-derived product (butanol), the titer was improved by overexpression of *ALD6*, *SEacs* and *PDC1* [13]. We therefore tested the effect of overexpressing genes *ALD6*, *SEacs*^*L641P*^ and *PDC1* in combination with multiple integration of *CaMCR* and ACC1^**^ on 3HP production. First, we constructed two chassis strains harboring an additional copy of either *ALD6*-*SEacs*^*L641P*^ under the control of *P*_*PGK1*_ and *P*_*TEF1*_ promoters, respectively, or of *ALD6*-*SEacs*^*L641P*^ in combination with *PDC1* under the control of *P*_*TEF1*_. These resulting strains were further transformed with pTY4-*CaMCR*–ACC1^**^. Overexpression of *ALD6*-*SEacs*^*L641P*^ improved the 3HP titer by 20 %, while combined overexpression of *ALD6*-*SEacs*^*L641P*^ and *PDC1* increased the titer by 80 % in both defined mineral and feed-in-time media (Fig. 2). Furthermore, gene copy number of *CaMCR*–ACC1^**^ in different strains was determined by qPCR, and we detected similar copies of both genes (3–4 copies) in all the constructed strains, which is in consistent with the copy number observed in strain carrying TY4-*CaMCR*–ACC1^**^ from the previous section. These results clearly show that enhancing the availability of intracellular acetyl-CoA had additive effects on 3HP production when combined with multiple copies of the *CaMCR* and ACC1^**^.

### Improvement of 3HP production by engineering the redox metabolism

**Fig. 3:**
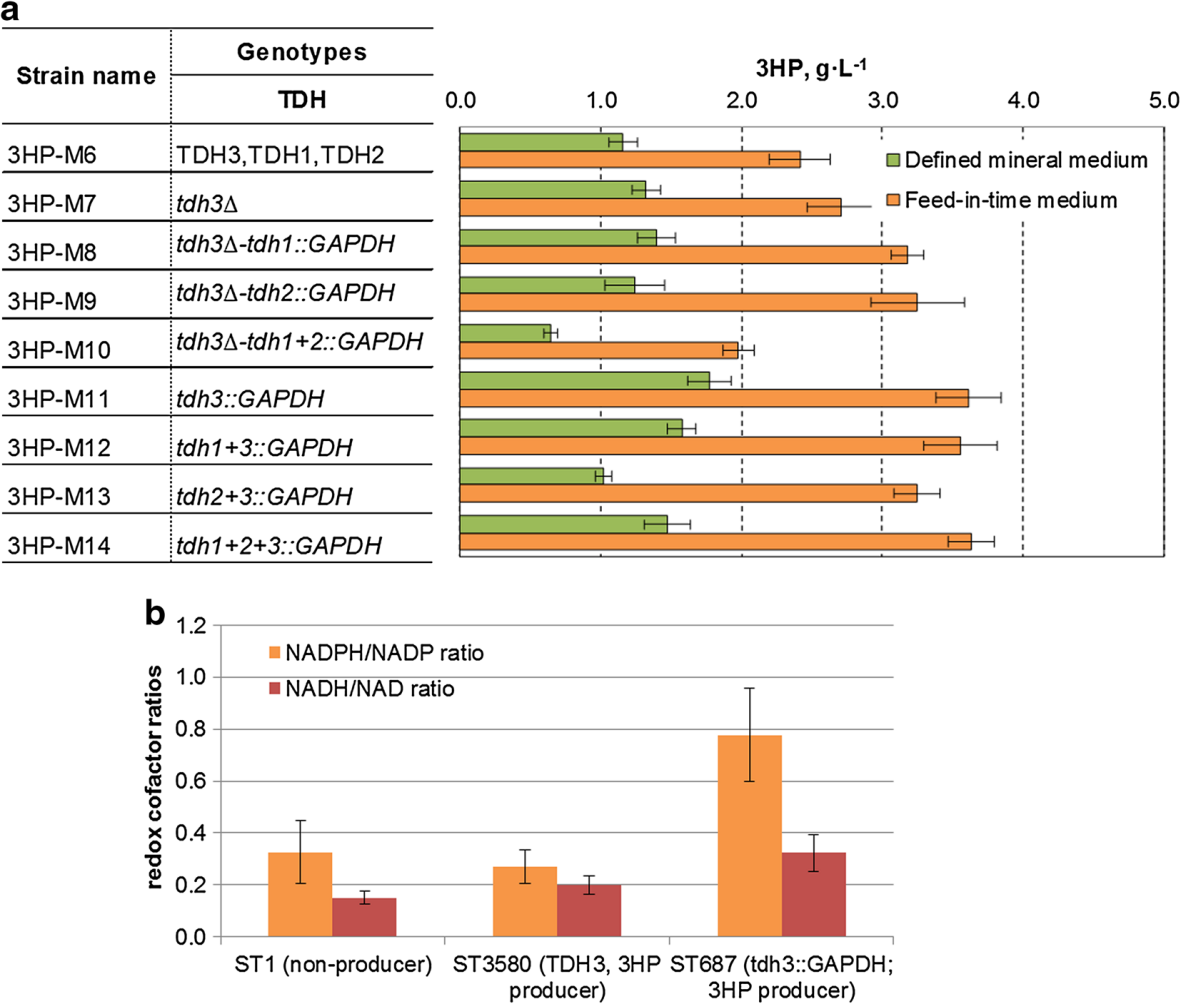
Production of 3HP in *S. cerevisiae* strains with cofactor engineering. **a** 3HP titer in the recombinant strains grown in defined mineral or feed-in-time media. *Error bars* represent the standard deviations in 12 biological replicates. **b** The redox cofactor ratios in the 3HP producer strains grown in defined mineral medium for 24 h. Displayed are the average values ± standard deviations from biological triplicates. All the constructed strains carried the overexpression of *ALD6*, *SEacs* ^*L641P*^, *PDC1*, and TY4-*CaMCR*–*ACC1* ^**^

*CaMCR* catalyzed reduction of malonyl-CoA to 3HP requires two NADPH. Therefore, the engineered pathway towards 3HP has a substantial need for this redox equivalent. Consequently, a yeast host with elevated NADPH availability is preferred. Different approaches have previously been reported to improve the supply of NADPH in *S. cerevisiae*. Heterologous expression of non-phosphorylating NADP-dependent glyceraldehyde-3-phosphate dehydrogenase (GAPN, EC 1.2.1.9) has been shown to improve bioethanol and 3HP production and minimize glycerol synthesis [6, 14, 15]. Another approach was heterologous expression of NADP-dependent glyceraldehyde-3-phosphate dehydrogenase (NADP-GAPDH, EC 1.2.1.13) from *Kluyveromyces lactis* (*KlGAPDH*), applied to improve xylose fermentation [13]. In addition, replacing the native *E. coli* NAD-GAPDH with NADP-GAPDH from *Clostridium acetobutylicum* resulted in significant improvements in lycopene and epsilon-caprolactone productivities [16]. In this study, we decided to overexpress a NADP-GAPDH gene from *C. acetobutylicum* (*CaGAPDH*) as this enzyme has higher affinity to NADP than *KlGAPDH* [16]. Verho and coworkers reported that *KlGAPDH* has equal kinetics for both NADP and NAD [17]. Additionally, we tried to reduce the potential futile cycle between the endogenous GAPDH and the introduced GAPDH by lowering the level of endogenous GAPDH activity. The NADP-dependent GAPDH was expressed in yeast strains, in which one, two or three of the endogenous NAD-dependent glyceraldehyde-3-phosphate dehydrogenase genes *TDH1, TDH2* or *TDH3* were deleted and/or exchanged with the coding sequence of GAPDH. By exchanging the coding sequence, we ensured that the introduced GAPDH had the same expression profile as the endogenous NAD-dependent GAPDH. Eight different combinations were made according to Additional file 1: Table S3. Into each of these eight strains and the reference strain we integrated *ALD6*, *SEacs*^*L641P*^, *PDC1*, and TY4-*CaMCR*–ACC1^**^. In both tested media, there was significant increase in 3HP titer for the strains, in which *TDH3* was replaced with *CaGAPDH* (*tdh3::CaGAPDH*). The effects of additional modifications to this replacement were minor (Fig. 3a). The strains without cofactor engineering ‘*TDH3*’ produced 1.2 ± 0.1 and 2.4 ± 0.2 g L^−1^ 3HP in defined mineral and feed-in-time media, respectively. The ‘*tdh3::CaGAPDH*’ strains gave 1.8 ± 0.2 and 3.6 ± 0.2 g L^−1^ 3HP in defined mineral and feed-in-time media, respectively. Furthermore, the ratio between 3HP and glycerol was nearly twofold higher for the ‘*tdh3::CaGAPDH’* strains grown in defined mineral medium compared to the strain without cofactor engineering ‘*TDH3*’ (Additional file 1: Fig. S2). On the other hand, the change in 3HP/glycerol was less pronounced when the strains were cultivated in feed-in-time medium. This was also expected because under carbon-limited conditions more carbon enters the pentose phosphate (PP) pathway, effectively resulting in higher NADPH production [18]. The best isolates among the strains without cofactor engineering ‘*TDH3*’ and ‘*tdh3::CaGAPDH*’ strains were named ST3580 and ST687, respectively. The intracellular concentrations of redox cofactors in ST3580, ST687 and non-producing strain (ST1) were further investigated. The 3HP-producing strain ST3580 strain had slightly lower NADPH/NADP ratio than the non-producing strain. By engineering the NADPH cofactor in strain ST687, the ratio between NADPH and NADP was significantly increased (Fig. 3b; Additional file 1: Fig. S3). Interestingly, the NADH/NAD ratio increased as well, which may explain why this strategy did not eliminate glycerol production. We also measured the μ_max_ of the three tested strains. ST687 had the lowest μ_max_ of 0.12 h^−1^, whereas μ_max_ of 0.16 h^−1^ was observed in ST3580. The decrease in the specific growth rate in *Δtdh3* strain was also observed earlier [19].

### Production of 3HP in controlled fed-batch fermentation

**Fig. 4:**
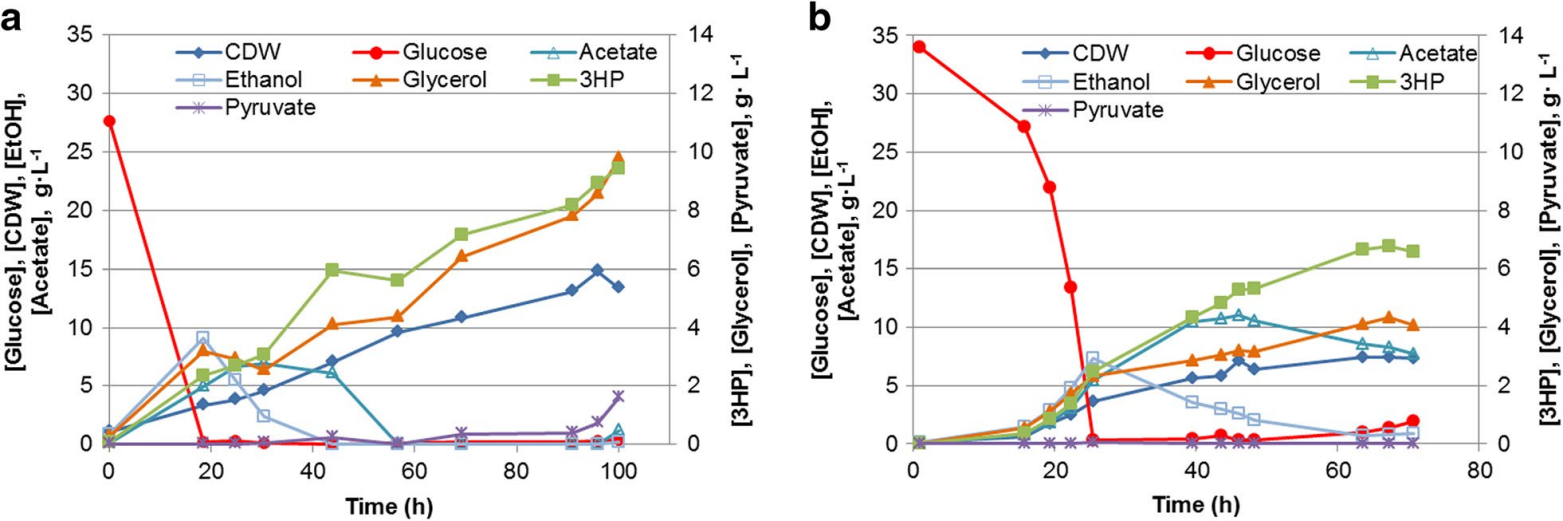
Fed-batch fermentation of the best 3HP-producing strain ST687. Aerobic fed-batch fermentations were carried out under **a** C-limited conditions and **b** N/C-limited conditions. The cultivations were carried out in triplicates (Additional file 1: Fig. S4); here representative graphs are shown

The best 3HP-producing yeast strain ST687 was cultivated under two different fermentation conditions: carbon-limited fed-batch (C) and nitrogen- and carbon-limited fed-batch (N/C). The results are summarized in Table 1, Fig. 4 and in Additional file 1: Fig. S4. ST687 strain produced up to 6.9 ± 1.0 and 9.8 ± 0.4 g L^−1^ 3HP in N/C-limited and C-limited fed-batch fermentations, respectively. This is a significant increase over the 3HP concentrations via malonyl-CoA pathway in *S. cerevisiae* reported earlier [6]. The 3HP/glycerol ratio observed in the C-limited fed-batch cultivations was similar to that observed in microtiter plate cultivations with feed-in-time medium described above. In N/C-limited fed-batch, the specific yield of 3HP and 3HP/glycerol ratio were slightly higher than under C-limitation. The final titer however was lower due to the low biomass concentration. Furthermore, higher acetate accumulation was also observed. Nitrogen limitation has been reported to result in higher lipid accumulation, which requires acetyl-CoA and NADPH [20]. Increased production of acetyl-CoA and NADPH under nitrogen limitation could explain the increased 3HP and acetate production and improved 3HP/glycerol ratio.

**Table 1:** 3HP yields and titers obtained during fed-batch cultivations

	C-limited	N/C-limited
Titer (3HP) (g L^−1^)	9.83 ± 0.43	6.93 ± 1.02
Volumetric production rate in fed-batch phase (g L^−1^ h^−1^)	0.09 ± 0.01	0.14 ± 0.02
Specific yield (g g^−1^ DW)	0.69 ± 0.05	0.85 ± 0.04
Overall yield, % C-mol C-mol^−1^ glucose (%)	13 ± 1	14 ± 1.8
3HP/glycerol ratio	0.85 ± 0.09	1.57 ± 0.05

### Transcriptome analysis of 3HP-producing *S. cerevisiae* strain

**Fig. 5:**
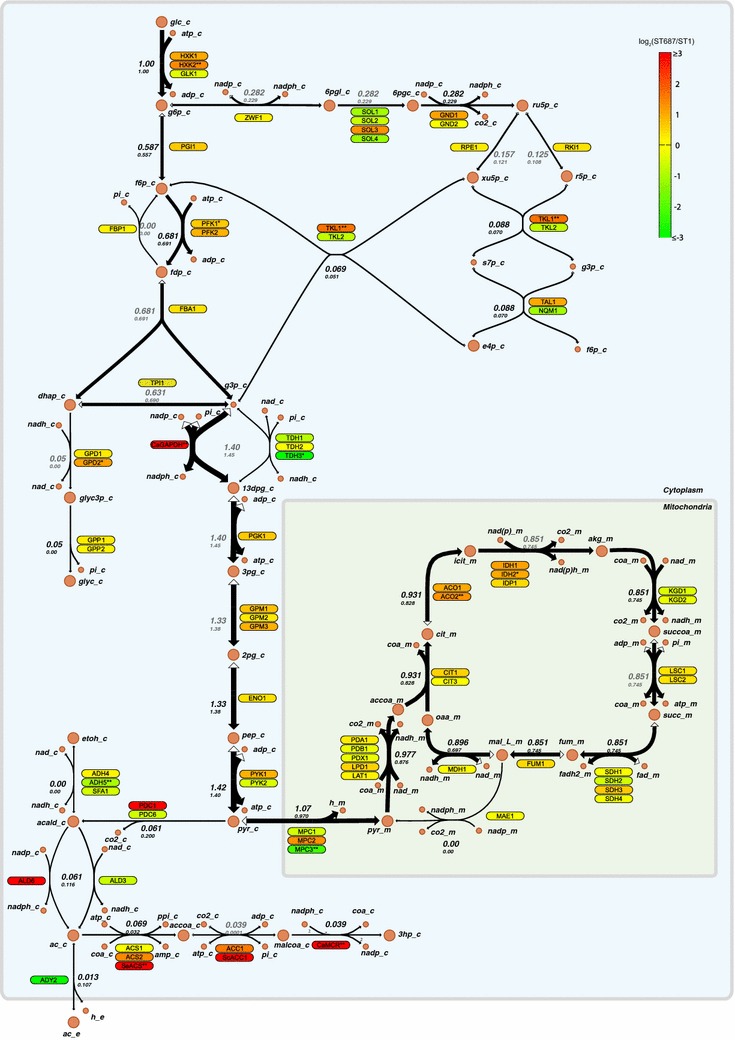
Transcriptome and metabolic flux analyses in the central carbon metabolism in ST687 and ST1 (non-producer) strains. The differentially expressed genes in ST687 compared with ST1 are highlighted with color. All the fluxes were normalized to the glucose uptake flux (set as 1). *The values in the first and second lines* correspond to the flux distributions in ST687 and ST1 strains, respectively. *Black numbers* are fluxes calculated from ^13^C analysis and *grey numbers* are predicted fluxes from the genome-scale model. *glc* glucose, *g6p* glucose-6-phosphate, *6pgl* d-6-phospho-glucono-δ-lactone, *6pgc* 6-phospho-d-gluconate, *ru5p* ribulose-5-phosphate, *xu5p* xylulose-5-phosphate, *s7p* sedoheptulose-7-phosphate, *e4p* erythrose-4-phosphate, *r5p* ribose-5-phosphate, *f6p* fructose-6-phosphate, *fdp* fructose-1,6-diphosphate, *g3p* glyceraldehyde-3-phosphate, *dhap* dihydroxy-acetone phosphate, *glyc3p* glycerol-3-phosphate, *glyc* glycerol, *13dpg* 1,3-diphosphateglycerate, *3pg* 3-phosphoglycerate, *2pg* 2-phosphoglycerate, *pep* phosphoenolpyruvate, *pyr* pyruvate, *acald* acetaldehyde, *etoh* ethanol, *acc* acetate, *accoa* acetyl-CoA, *malcoa* malonyl-CoA, *3hp* 3-hydroxypropionic acid, *cit* citrate, *icit* isocitrate, *akg* α-ketoglutarate, *succoa* succinyl-CoA, *succ* succinate, *fum* fumarate, *mal* malate, *oaa* oxaloacetate

Genome-wide transcriptome analysis has been used to explore the cellular response to different perturbations, e.g. altered growth conditions or genetic changes. In this study, we performed transcriptome analysis using RNA sequencing on the two *S. cerevisiae* strains, the non-producing strain ST1 and the best 3HP-producing strain ST687, in order to explore the change in gene expression profile in response to the engineered 3HP production. When comparing the ST1 and ST687 strains, 474 genes with significant changed mRNA levels (q ≤ 0.05) were identified. Among these 474 genes, 326 genes showed a change of more than twofold (Additional file 2: Table S6). Apart from expected genes that were purposely over-expressed in ST687 (*CaMCR*, *ACC1*, *PDC1*, *ALD6*, *ACS*, *CaGAPDH*), several genes involved in the PP pathway (*SOL3*, *GND1*, *TKL1*, *TAL1*) and tricarboxylic acid (TCA) cycle (*MPC2, IDH1*, *IDH2*, *ACO1*, *ACO2*) were up-regulated (Fig. 5). By contrast, the expression of *ZWF1*, the rate limiting reaction in the entire PP pathway, was not affected by redox perturbations. Increased expression of PP pathway genes and of isocitrate dehydrogenase genes *IDH1* and *IDH2* may lead to improved NADPH availability in the cytosol for 3HP production. The upregulation of several PP pathway genes (i.e., *SOL3*, *GND1*, *TKL1*, *TAL1*) in response to an increase in NADPH demand has previously been reported [21]. In addition, a handful of genes related to redox metabolism were either down-regulated (*FDH1*, *NDE2*, *OYE2*, *OYE3*, *ADH5*) or up-regulated (*GPD2*, *GSH1*, *BNA4*). We propose that the changes in the expression of genes involved in the redox metabolism are due to the NADPH demand for 3HP biosynthesis. Additionally, some genes in the glycolytic pathway (*HXK1*, *HXK2*) were also up-regulated in ST687. Furthermore, several genes belonging to biotin biosynthesis/biotin related proteins were up-regulated such as *BIO3*, *BIO4*, *BIO5* and *PET8*. This could be due to overexpression of a gene encoding for Acc1p, which uses biotin as cofactor. Significant changes in transcription of genes related to amino acid synthesis and transport were also observed (up-regulated: *HOM3*, *SER3*, *GLN1*; and down-regulated: *ADY2*, *ALT2*, *BAP3*, *VBA5*, *GNP1* and *AGP1*). The increase in the expression of genes involved in amino acid synthesis, particularly those involved in the NADPH-consuming methionine and lysine pathway and serine biosynthesis, might be due to an indirect response to the higher NADPH availability [21].

## ^13^C Metabolic flux analysis of 3HP producing *S. cerevisiae*

Metabolic flux analysis combined with genome-scale modeling is a powerful tool for studying the global response of the cellular metabolism to environmental or genetic changes and for identifying the mechanisms involved in re-routing the metabolic fluxes. In this study, we performed ^13^C metabolic flux analysis on the two *S. cerevisiae* strains, non-producing strain (ST1) and the best 3HP-producing strain (ST687), to gain a better understanding of the influence of 3HP biosynthesis on the flux distribution. Both strains were cultivated in carbon-limited chemostats at 0.04 h^−1^ either with 100 % 1-^13^C labeled glucose or a mixture of 20 % U–^13^C-labelled and 80 % natural-labelled glucose. After 4–5 retention times, the steady-state culture was harvested and the labelling was analyzed by GC–MS and the fluxes were calculated using a central carbon model in ^13^C flux software [22] (redox balance was not accounted for in the model, the calculations were made only based on carbon-balance and labelling pattern). The flux values obtained from ^13^C analysis were used as constraints for a genome-scale model, where redox balance was enforced and the residual fluxes were calculated. The fluxes in the central carbon metabolism in the ST687 and ST1 strains are presented in Fig. 5 and Additional file 3: Table S7. Notably, the biomass yield on glucose was significantly lower for the producing strain (0.40 g DW g glucose^−1^) in comparison to 0.50 in ST1 strain. The decrease cannot be accounted for by 3HP flux alone (0.02 g 3HP g glucose^−1^), and hence there must also be more carbon dioxide produced by the ST687. The flux through the PP pathway and TCA cycle was increased by correspondingly 23 and 12 % in the ST687 strain, which is consistent with transcriptional analysis showing up-regulation of several genes in the PP and TCA pathways in the ST687 strain.

The flux from acetate into acetyl-CoA was increased in ST687, which might be due to a combination of overexpression of *ACS* and down-regulation of acetate transporter *ADY2*, whereas higher acetate secretion was observed in the ST1 strain. In ST687, most of the acetate was converted into 3HP and biomass. On the other hand, the flux from pyruvate into acetate dropped to nearly half in ST687 even though the corresponding genes were overexpressed.

The combined data on physiological characterization, transcriptome and flux distribution revealed several unexpected consequences of the undertaken metabolic engineering strategies. Firstly, in spite of our attempts to provide more NADPH by exchanging a native NAD-dependent glyceraldehyde-3-phosphate dehydrogenase with a heterologous NADP-dependent version, the redox imbalance has not been completely resolved. Strain ST687 continued to produce glycerol in cohort with 3HP production. Even more surprisingly, the two major pathways for NADPH regeneration, PP pathway and isocitrate dehydrogenases, were transcriptionally upregulated and carried higher flux in the engineered strain. The ST687 strain had increased ratios of NADPH/NADP, but also of NADH/NAD. These findings underline the complexity and high connectivity of the redox metabolism and the difficulties associated with engineering of the redox metabolism. Secondly, we found that the flux from pyruvate towards acetate actually dropped 2.5-fold, against expectations. The transcriptome data showed that all the genes in the pyruvate dehydrogenase by-pass pathway were highly upregulated and the flux from acetate to acetyl-CoA increased, but there was obviously a limitation in pyruvate conversion into acetate.

These findings have implications both on the direction of the next metabolic engineering efforts and on the process considerations. The chosen carbon-limited feeding strategy limits the overflow metabolism and hence leads to very low flux to acetate. If a batch-type fermentation mode is chosen instead, then a significant part of carbon will be lost to ethanol and hence ethanol formation must be prevented. This can be achieved either by deletion of the numerous native alcohol dehydrogenases [23] or by deletion of pyruvate decarboxylases [24], both very challenging strategies, the latter would also require installing a different pathway for cytosolic acetyl-CoA supply for 3HP, e.g. a recently described cytosolic pyruvate dehydrogenase complex [25]. On the other hand, using batch cultivation would lead to low flux via PP pathway, which may reduce the supply of NADPH cofactor for 3HP production. To avoid glycerol formation, deletion of *GPD2* can be considered [26], however if the redox imbalance is not restored, the strain will suffer from reduced growth rate and viability and will have decreased tolerance to osmotic stress. For further metabolic engineering, many factors must be taken into consideration and preferably multiple potential strategies should be investigated. Dynamic regulation of 3HP expression and growth-essential genes may allow decoupling the growth and production phases and hence lead to higher product titer, rate and yield.

Our study yet again demonstrated that in strain engineering it is not sufficient to look at the resulting product titers, but one needs more in-depth characterization of the cellular physiology and cellular components and fluxes in order to follow and push forward the strain development.

## Conclusions

Here we engineered and optimized 3HP production in *S. cerevisiae* via the malonyl-CoA pathway. Integration of multiple copies of biosynthetic genes, overexpression of precursor supply genes, and engineering the redox cofactor all improved the 3HP production, resulting in a strain able to produce 3HP at a titer of 9.8 ± 0.4 g L^−1^ with a yield of 13 % C-mol C-mol^−1^ glucose after 100 h at pH 5 in C-limited fed-batch cultivation. This strain represents a platform for further optimization of 3HP production and hence an important step towards potential commercial, bio-based production of 3HP.

## Methods

### Strains and maintenance

*Saccharomyces cerevisiae* CEN.PK strains were obtained from Peter Kötter (Johann Wolfgang Goethe-University Frankfurt, Germany). Recombinant yeast strains were selected on synthetic drop-out agar plates (Sigma–Aldrich) at 30 °C. Cultivation of the recombinant strains was performed in defined mineral medium or synthetic fed-batch medium Sc.syn-1000 (M2P labs GmbH, Germany). The defined mineral medium contained per liter: 7.5 g (NH_4_)_2_SO_4_, 14.4 g KH_2_PO_4_, 0.5 g MgSO_4_·7H_2_O, 22 g dextrose, 2 mL trace metals solution, and 1 mL vitamins. The salts were dissolved in water, pH was adjusted to 6 and the solution was autoclaved. Glucose solution was sterilized separately. Glucose solution and vitamins solution were added to the autoclaved salts solution. The trace metals solution contained per liter: 4.5 g CaCl_2_·2H_2_O, 4.5 g ZnSO_4_·7H_2_O, 3 g FeSO_4_·7H_2_O, 1 g H_3_BO_3_, 1 g MnCl_2_·4H_2_O, 0.4 g Na_2_MoO_4_·2H_2_O, 0.3 g CoCl_2_·6H_2_O, 0.1 g CuSO_4_·5H_2_O, 0.1 g KI, and 15 g EDTA. The trace metals solution was prepared by dissolving all the components except EDTA in 900 mL ultra-pure water at pH 6. The solution was then gently heated and EDTA was added. In the end, the pH was adjusted to 4, and the solution volume was adjusted to 1 L and autoclaved (121 °C in 20 min). This solution was stored at 4 °C. The vitamins solution had per liter: 50 mg biotin, 200 mg *p*-aminobenzoic acid, 1 g nicotinic acid, 1 g Ca-pantotenate, 1 g pyridoxine–HCl, 1 g thiamine–HCl, and 25 g myo-inositol. Biotin was dissolved in 20 mL 0.1 M NaOH and 900 mL water is added. pH was adjusted to 6.5 with HCl and the rest of the vitamins was added. pH was re-adjusted to 6.5 just before and after adding m-inositol. The final volume was adjusted to 1 L and sterile-filtered before storage at 4 °C [10].

The chemicals were obtained, if not indicated otherwise, from Sigma–Aldrich. The 3-hydroxypropionic was purchased from Tokyo Chemicals Industry Co. (TCI).

*Escherichia coli* strains were cultivated on Luria–Bertani (LB) medium with appropriate antibiotic selection at 37 °C. *E. coli* transformants were selected on LB medium containing 100 µg mL^−1^ ampicillin.

### Plasmids construction

The primers, biobricks and plasmids used in this study are listed in Additional file 1: Tables S1, S2 and S3. The biobricks were assembled into episomal, integrative or multiple integrative plasmids using USER cloning [10]. DNA manipulations in *E. coli* were carried out according to standard procedures. The clones with correct inserts were identified by colony PCR and the plasmids were isolated from overnight *E. coli* cultures and confirmed by sequencing.

### Strains construction

All strains used in this study are listed in Additional file 1: Table S4. The integrative plasmids were *Not*I-linearized and transformed into *S. cerevisiae* cells using the lithium acetate transformation protocol [27]. The cells were selected on synthetic drop-out medium. The *tdh*-strains were constructed sequentially as indicated in the Additional file 1: Table S4. The linear PCR fragments used for transformation were constructed by combining USER, T4 ligation and PCR amplification. The final transformation fragments were made from 2 to 3 individual PCR fragments with matching USER tails, which were mixed with USER enzyme and incubated for 25 min at 37 °C followed by 20 min at 25 °C. After USER reaction T4 ligase was added and the mix was incubated for 5 min at room temperature. One micro-litre of this ligation mix was used as template for the final PCR reaction in order to amplify the whole assembled DNA fragment. The fragments were purified from gel and used for yeast transformation (100–300 ng per transformation).

All strains were verified by PCR using insert-specific oligos in combination with oligos specific to the regions outside the different recombination sites. The CreA-loxP-mediated selection marker loop-out was done as described previously [10]. The elimination of the selection marker was verified by PCR in addition to phenotypic tests.

### Cultivation in microtiter plates

At least six single colonies originating from independent transformants were inoculated in 0.5 mL synthetic drop-out liquid medium without uracil, histidine, and leucine (SC-ura-his-leu) in a 96-deep well microtiter plate with air-penetrable lid (EnzyScreen, NL). The plates were incubated at 30 °C with 250 rpm agitation at 5 cm orbit cast overnight. 50 µL of the overnight cultures were used to inoculate 0.5 mL defined mineral medium or synthetic fed-batch medium (feed-in-time medium), in a 96-deep well plate. Fermentation was carried out for 72 h at the same conditions as above.

At the end of the cultivation OD_600_ was measured as previously described [5]. The culture broth was spun down and the supernatant analyzed for metabolites concentration using HPLC. The variation in 3HP titer did not exceed 10 % between different batches of the same strain.

### Controlled fermentations

ST687 glycerol stock (0.3 mL) was inoculated in 150 mL SC-ura-his-leu medium in a 250-mL baffled shake flask and propagated at 30 °C with 250 rpm agitation for about 24 h. The culture was concentrated down to 50 mL by centrifugation at 4000× rpm for 2 min and used to inoculate 0.5 L defined mineral medium in 1 L-Sartorius reactor. Culture media and growth conditions were the same as described before [5]. The agitation rate was 800 rpm, the temperature was 30 °C, aeration was 1 L min^−1^ air and pH was maintained at 5.0 by automatic addition of 2 N NaOH. For fed-batch cultivations, the feed was started after the glucose was exhausted. For C-limited fed-batch cultivation, the feed contained per liter: 45 g (NH_4_)_2_SO_4_, 18 g KH_2_PO_4_, 3 g MgSO_4_·7H_2_O, 12 mL trace metals solution, 6 mL vitamins solution, 0.6 mL antifoam A, and 176 g dextrose. Dextrose was autoclaved separately, vitamins solution was sterile filtered and added to the feed after autoclavation. The initial feed rate was set to 5 g h^−1^ and 72 h after the feed start it was further increased to 15 g h^−1^. The total feed volume used per reactor was 0.5 L. For N/C-limited fed-batch cultivation, the feed contained per liter: 200 g dextrose as well as 6 mL trace metals solution and 3 mL vitamin solution. During the first 14 h of the feeding phase a feed rate of 1 g h^−1^ was applied, after that the feed rate was reduced to 0.6 g h^−1^. The reactors were sampled twice a day to measure biomass dry weight and metabolites. For metabolites analysis the sample was immediately centrifuged and the supernatant was stored at −20 °C until HPLC analysis. Continuous cultivation for transcriptome analysis was performed using mineral medium as described before [10], but 3 g L^−1^ of KH_2_PO_4_ was used instead of 14.4 g L^−1^. After glucose was exhausted, the feed was started at the dilution rate of 0.04 h^−1^. Once the steady-state has been established, the cultivation was allowed to proceed for three residence times and samples for metabolites analysis and transcriptome were withdrawn. The feed medium was the same for both batch and continuous phases.

### Analysis of metabolites and cell dry weight

Cell growth was determined by measuring the absorbance at 600 nm wavelength. The extracellular metabolites were quantified by HPLC as previously described [5]. Glucose, glycerol and ethanol were detected using a RI-101 Refractive Index Detector (Dionex). 3HP, pyruvate, succinate and acetate were detected with a DAD-3000 diode array detector at 210 nm (Dionex).

### qPCR analysis

The copy numbers of *CaMCR* and ACC1^**^ genes in the recombinant yeast strains overexpressing these genes were determined by qPCR. Genomic DNA from three isolates from each strain was isolated using ZR Fungal/Bacterial DNA MiniPrepTM kit (Zymo Reasearch Corporation) according to the manufacturer’s recommendation. qPCR analysis of gDNA was carried out in triplicate using Brilliant III Ultra-Fast SYBR^®^ Green QPCR Master Mix (Agilent Technologies) on a Stratagene Mx3005P (Agilent Technologies). The thermal cycling conditions were 95 °C, 10 min followed by 40 cycles of 95 °C for 20 s and 60 °C for 22 s, then 1 cycle of 95 °C for 1 min, 55 °C for 30 min, and 95 °C for 30 s. The gene copy numbers were measured relative to that of a housekeeping gene (*ALG9*). Primers used for qPCR are listed in Additional file 1: Table S1.

### Intracellular redox cofactors analysis

The strains were cultivated in 5 mL of SC-ura-his-leu in 15-mL plastic tubes at 30 °C with 250 rpm overnight. The overnight cultures were used to inoculate 50 mL mineral medium in 250 mL shake flasks to an initial OD of 0.05. For ST687 strain, an initial OD_600_ of 0.1 was used due to slow growth. The shake flasks were incubated at 30 °C with 250 rpm. After 24 h of incubation, the samples were withdrawn and quenched in 5 ml of cold methanol (−40 °C). The sample volume withdrawn was adjusted to give OD_600_ of 15 that is required for the analysis. The sample volume did not exceed 2 mL for any samples. The samples were immediately centrifuged at 4000 rpm, for 5 min. After the centrifugation, the supernatant was removed and the cell pellet was frozen in liquid nitrogen and stored at −80 °C before the analysis. All cultivations were done in biological triplicates. Each shake flask was sampled in five technical replicates. Intracellular metabolites were extracted by using boiling ethanol method and redox cofactors were analyzed by LC–MS/MS (for details on sample preparation and LC/MS analysis see Additional file 1: Supplementary methods).

## ^13^C-labelling experiments and analytical procedure (GC–MS)

In order to quantify the intracellular fluxes, a combination of metabolite balancing and isotope labeling experiments were applied for the reference and the engineered 3HP-producing strain.

Continuous cultivations were performed in mini-bioreactors with a working volume of 10 mL. The setup was based on the mini-scale reactor system described by [28]. Hungate tubes (16 mm × 125 mm, Dunnlab, Germany) with a butyl rubber septum were used as culture vessels. All tubes were fixed in a tempered water bath at 30 °C. Medium feed and fermentation broth efflux was realized by a multichannel peristaltic pump (IPC 12 Ismatec, Wertheim, Germany). To minimize the risk of back-contamination a fused silica capillary (ID 100 µm Optronis, Kehl, Germany) was used for feed supply. The harvest pump was connected to a needle (ID 0.8 mm, L 120 mm), which was positioned at the corresponding height of 10 mL to maintain a constant liquid volume. The efflux rate was set to 0.5 mL h^−1^ and always exceeded the feed rate. A third multichannel pump (Ecoline VC-MS/CA8-6 Ismatec, Wertheim, Germany) actively pumped humidified air into the mini-reactors via a submerged needle (ID 1 mm, L 200 mm). The aeration rate was set to 2 vvm. Off-gas was directed via a short needle (ID 1.2 mm, L 80 mm) to an O_2_/CO_2_ gas analyzer (BlueInOne, BlueSens, Herten, Germany). Additional mixing of the culture liquid was realized with magnetic stirrer discs at 900 rpm. A fourth needle (ID 0.8 mm, L 120 mm) was used as sampling port.

The cultivation was started with an initial batch fermentation in minimal medium (pH 5, buffered with 100 mM potassium hydrogen phthalate) with 10 g L^−1^ glucose. After cells reached stationary phase, indicated by the drop of the off-gas CO_2_ signal, feed and harvest pumps were started and the fermentation was switched to continuous mode. The dilution rate was set to 0.04 h^−1^ and the supplied feed medium was equal to the batch medium. For ^13^C labeling experiments 100 % 1-^13^C labeled glucose (99 % purity, Euriso-Top GmbH, Saarbrücken, Germany) or a mixture of 20 % (n/n) U–^13^C glucose (99 % purity, Euriso-Top GmbH, Saarbrücken, Germany) and 80 % (n/n) naturally labeled glucose were used. Metabolic steady-state conditions were checked by stability of the CO_2_ and O_2_ signals in the off-gas and optical density measurements. Samples for HPLC measurements were taken for quantification of residual glucose, 3HP and metabolic byproducts. After 4–5 residence times, samples of 0.3 mg CDW were taken from the culture broth for gas chromatography–mass spectrometry (GC–MS) analysis of proteinogenic amino acids. The cells were harvested by centrifugation, washed once with sterile water and hydrolyzed in 500 mL 6 M HCl at 105 °C for 6 h. The hydrolysate was dried in a heating block at 85 °C. The free amino acids were resuspended in 30 µL acetonitrile and 30 µL N-methyl-N-(tert-butyldimethylsilyl)-trifluoroacetamide (MBDSTFA, CS–Chromatographie Service GmbH, Langerwehe, Germany) for 1 h at 85 °C. The samples were cooled down to room temperature and analyzed immediately by GC–MS.

Gas chromatographic separation was performed on a Thermo Scientific (Thermo Scientific, Waltham, MA, USA) Trace GC Ultra equipped with a Thermo Scientific AS 3000 autosampler and a Thermo Scientific (Thermo Scientific, Waltham, MA, USA) TraceGOLD TG-5SilMS capillary column (length, 30 m; inner diameter, 0.25 mm; film thickness, 0.25 µm). Separation of amino acids was performed at a constant flow rate of 1 mL helium min^−1^. A sample volume of 1 µL was injected into a split/splitless injector at 270 °C with a split ratio of 1:15. The temperature of the GC oven was kept constant for 1 min at 140 °C and afterwards increased with a gradient of 10 °C min^−1^ to 310 °C and again kept constant for 1 min. Mass spectrometry analysis was performed on a Thermo Scientific (Thermo Scientific, Waltham, MA, USA) ISQ single quadrupole mass spectrometer. The temperatures of the transfer line and the ion source were set to 280 °C. Ionization was performed by electron impact (EI) ionization at 70 eV. GC–MS raw data were analyzed with Xcalibur.

## ^13^C-constrained flux analysis

FiatFlux [22] was used to calculate metabolic flux ratios and net intracellular flux distributions as described in [29, 30]. The stoichiometric model of *S. cerevisiae*’s central carbon metabolism, described in [29], was extended with either of the 3HP biosynthesis pathways and constrained with the specific extracellular rates of biomass production (µ), glucose uptake rate, 3HP synthesis rate, and formation of the metabolic byproducts glycerol, acetate and pyruvate. The network was further constrained with linear equations derived from the eight metabolic flux ratios: fraction of cytosolic oxaloacetate originating from cytosolic pyruvate, fraction of mitochondrial oxaloacetate derived through anaplerosis, fraction of phosphoenolpyruvate originating from cytosolic oxaloacetate, fraction of serine derived through glycolysis, upper and lower bounds of mitochondrial pyruvate derived through malic enzyme, contribution of glycine to serine biosynthesis, and contribution of serine to glycine biosynthesis. Mass balances for CO_2_, O_2_, ATP and NADPH were not used for flux calculation.

### Transcriptome analysis

Samples for transcriptome sequencing (RNAseq) were taken after three residence times of the steady state continuous cultivation in 1L-bioreactors. Sampling procedure and total RNA extraction were performed as described before [31].

The sequencing libraries were prepared in triplicates using a TruSeq Sample Preparation kit (Illumina Inc., San Diego CA) and were pooled together prior to sequencing. An average cDNA library size was determined using the Agilent DNA 1000 kit on an Agilent 2100 Bioanalyzer. Libraries were normalized and pooled in 10 mM Tris–Cl, pH 8.0, plus 0.05 % Tween 20 to the final concentration of 10 nM. Denaturated in 0.2 N NaOH, 10 pm pool of 6 libraries in 600 μL ice-cold HT1 buffer was loaded onto the flow cell provided in the MiSeq Reagent kit v2 300 cycles and sequenced on a MiSeq^®^ (Illumina Inc., San Diego CA) platform with a paired-end protocol and read lengths of 151 nucleotides.

TopHat (2.0.13) and Cufflinks (2.2.1) suite was used for RNAseq-based differential gene expression analysis as described by Trapnell et al. 2012 [32]. Reference genome and annotations for CEN.PK113-7D strain were retrieved from *Saccharomyces* Genome Database (SGD).

### Mathematical modeling and genome scale flux analysis

Fluxes were determined through computer assisted metabolic engineering and optimization (CAMEO) toolbox (doi: 10.5281/zenodo.18400) using parsimonious flux balance analysis (pFBA). A modified iTO977 yeast model [33] was used, for which the fluxes were bound to the ^13^C-determined flux ranges where available (for details on the modifications see Additional file 1: Supplementary methods). Escher [34] was used to make flux map figures.

## Additional files

10.1186/s12934-016-0451-5 Supplementary methods and supplementary results.
10.1186/s12934-016-0451-5 Transcriptome analysis of ST687 compared to non-producing strain ST1.
10.1186/s12934-016-0451-5 ^13^C Flux analysis of ST687 and non-producing strain ST1.

## Abbreviations

3HP: 3-hydroxypropionic acid
MCR: malonyl-CoA reductase
ACC1: acetyl-CoA carboxylase
PDC1: pyruvate decarboxylase
ALD6: aldehyde dehydrogenase
acs: acetyl-CoA synthase
GAPN: non-phosphorylating NADP-dependent glyceraldehyde-3-phosphate dehydrogenase
GAPDH: NADP-dependent glyceraldehyde-3-phosphate dehydrogenase
PP pathway: pentose phosphate pathway
TCA cycle: tricarboxylic acid cycle
C-limited fed-batch: carbon-limited fed-batch
N/C-limited fed-batch: nitrogen and carbon- limited fed-batch
Luria–Bertani medium: LB medium
