# Supplementary material for: Engineering and systems-level analysis of Saccharomyces cerevisiae for production of 3-hydroxypropionic acid via malonyl-CoA reductase-dependent pathway
Source: Microb Cell Fact. 2016 Mar 15;15:53. doi: 10.1186/s12934-016-0451-5 (PMC4791802; doi:10.1186/s12934-016-0451-5)
Supplement: Supplementary file 1 — 10.1186/s12934-016-0451-5 Supplementary methods and supplementary results. [file 12934_2016_451_MOESM1_ESM.docx]

**Supplementary information**

# Engineering and systems-level analysis of *Saccharomyces cerevisiae* for production of 3-hydroxypropionic acid via malonyl-CoA reductase-dependent pathway

Kanchana R. Kildegaard^a*^, Niels B. Jensen^a*,^^[[1]](#footnote-1)^, Konstantin Schneider^a^, Eik Czarnotta^b^, Emre Özdemir^a^, Tobias Klein^a^, Jérôme Maury^a^, Birgitta E. Ebert^b^, Hanne B. Christensen^a^, Yun Chen^c,d^, Il-Kwon Kim^c,d,^^[[2]](#footnote-2)^, Markus J. Herrgård^a^, Lars M. Blank^b^, Jochen Forster^a^, Jens Nielsen^a,c^, Irina Borodina^a#^

^a^The Novo Nordisk Foundation Center for Biosustainability, Technical University of Denmark, Kogle Allé 6, 2970 Hørsholm, Denmark, ^b^Institute of Applied Microbiology, RWTH Aachen University, Worringer Weg 1, 52056 Aachen, Germany, ^c^Department of Biology and Biological Engineering, Chalmers University of Technology, Kemivägen 10, SE-41296 Gothenburg, Sweden, ^d^The Novo Nordisk Foundation Center for Biosustainability, Chalmers University of Technology, Kemivägen 10, SE-41296 Gothenburg, Sweden.

**Additional file 1. Supplementary methods and supplementary results**

## Intracellular redox cofactors analysis

***Extraction***

Tubes containing boiling ethanol were prepared by placing 5 mL 75% ethanol in a preheated water bath at 95 °C for 5 min. The extraction was done quickly by adding 5 ml boiling ethanol to the cell pellet and immediately vortexed. The samples was placed in the water bath (95 °C) for 3 min. Hereafter they were transferred to dry ice with ethanol to cool down as fast as possible. The samples were spun down at max speed, 5 min, 4°C, and then dried down to dryness in a spec vac. The samples were the re-dissolve in 100 μL 20 mM ammonium formate, adjusted to pH 7.5 with ammonia hydroxide. Then the samples were vortex, spun down at max speed, 5 min, 4°C and finally transferred to LC-MS vials for analysis. The samples were analyzed by LC-MS/MS immediately hereafter.

***LC/MS analysis***

The LC-MS/MS system consisted of a CTC autosampler module, a high pressure mixing pump and a column module (Advance, Bruker, Fremont, CA, USA). The injection volume was 1 µL. The chromatography was performed on a hypergold PFP column, 150mm × 2.1 mm, 1.9 µm pore size (Thermoscientific, MA, USA). In front of the separation column was a 0.5 µ depth filter and guard column, the filter (KrudKatcher Classic, phenomenex). Column temperature was at 30 °C and the flow rate was set at 300 µL·min^-1^. Eluent A: 20 mM ammonium acetate pH adjusted to 7.5 with ammonium hydroxide in milliQ water. Eluent B: Acetonitrile. Calibration standards were prepared in eluent A, and the concentration was in the range from 1.25 µg·mL^-1^ -500 µg·mL^-1^.

The MS/MS detection was performed on a EVOQ triple quadrupole instrument (Bruker, Freemont, CA, USA) equipped with an atmospheric pressure ionization (API) interface. The mass spectrometer was operated with electrospray in the positive ion mode (ESI+). The spray voltage was set to 4500 V. The con gas flow was 20 L·h^-1^, and the cone temperature was set at 350°C. The heated probe gas flow was set at 50 L·h^-1^ with a temperature of 350°C. Nebulizer flow was set at 50 L·h^-1^, and the exhaust gas was turned on. Argon was used as collision gas at a pressure of 1.5 mTorr. Detection was performed in multiple reacting monitoring (MRM) mode. The transition for the investigated compounds was optimized with regard to collision energy. The MRM transitions are given in Table S5.

**Yeast model.**

***Model adjustments and modifications:***

TDH2 reaction is added (Glyceraldehyde 3-phosphate + NADP -> 3-phospho-D-glyceroyl phosphate + NADPH).

CaMCR reaction is added (Malonyl-CoA + 2 NADPH --> 3-hydroxypropionic acid + CoA + 2 NADP).

Transport across membrane and export of 3-hydroxypropionic acid is allowed.

Reaction U98 is blocked and replaced with ALD22x reaction (3-Aminopropanal +NAD--> B-alanine + NADH) based on [ref:PMC1462426].

Reaction U214's lower bound is set to 1 mmol/gDW/h to account for non-growth associated maintenance.

Catalytically inactive YDR111C (*ALT2*) reaction is blocked. [ref:PMC3458083]

**Supplementary Figures**

**
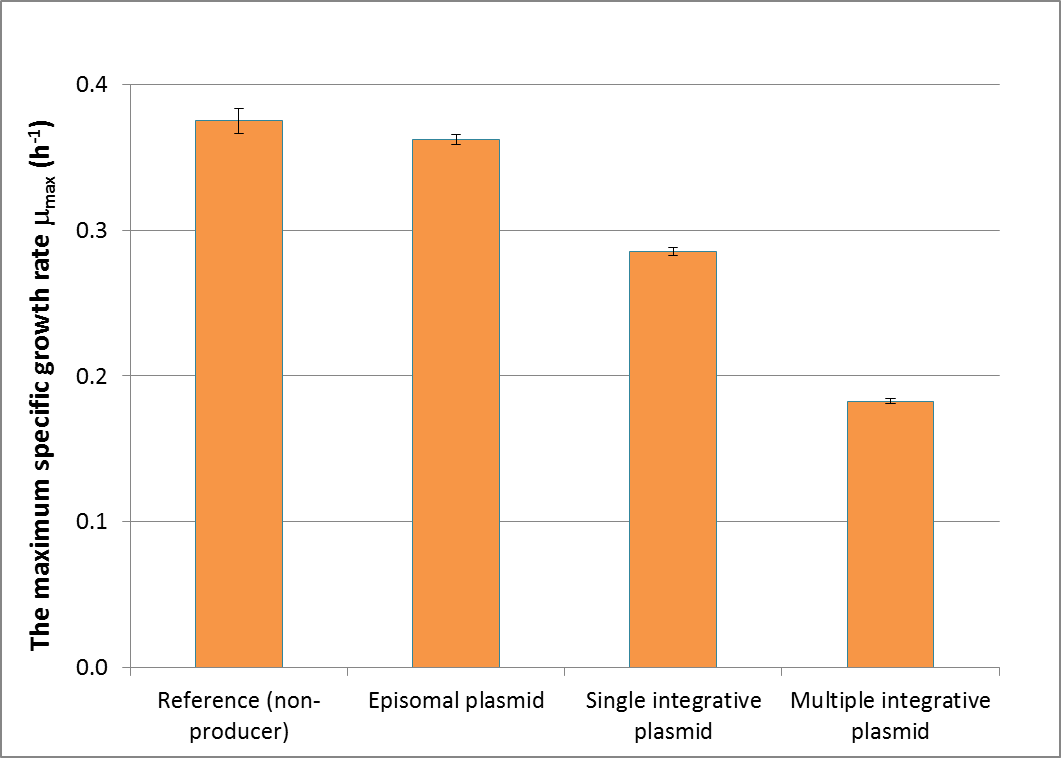
**

**Figure S1.** The maximum specific growth rate (μ_max_) of the recombinant strains grown in defined mineral medium in 96-well plate. Data are mean and + standard deviations from six independent colonies from each strains. The experiments were performed in triplicates.


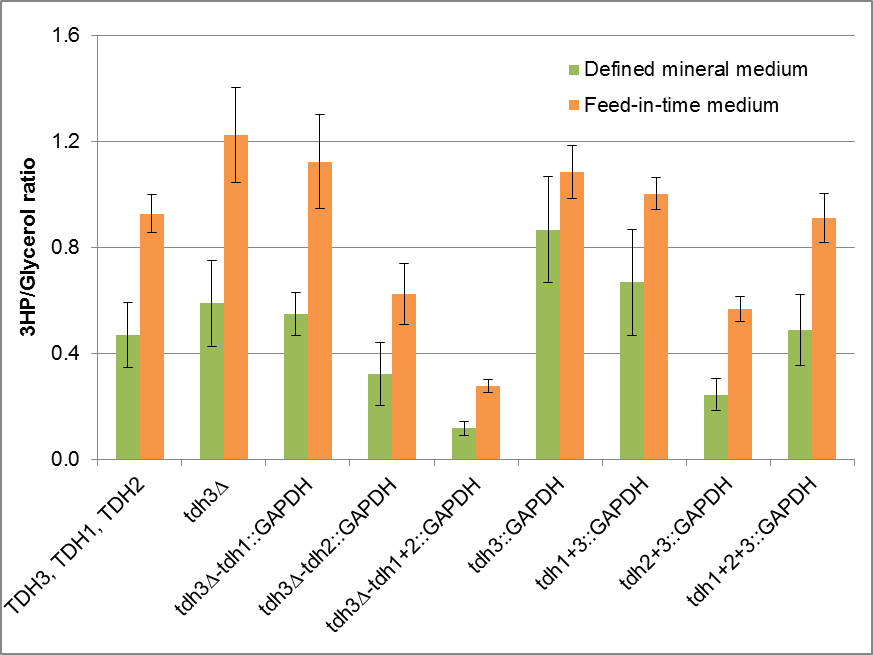


**Figure S2.**The ratio of 3HP/Glycerol in the recombinant strains grown in defined mineral or feed-in-time media. Data are mean and + standard deviation from six independent colonies from each strains. The cultivations were performed in biological triplicates.

**
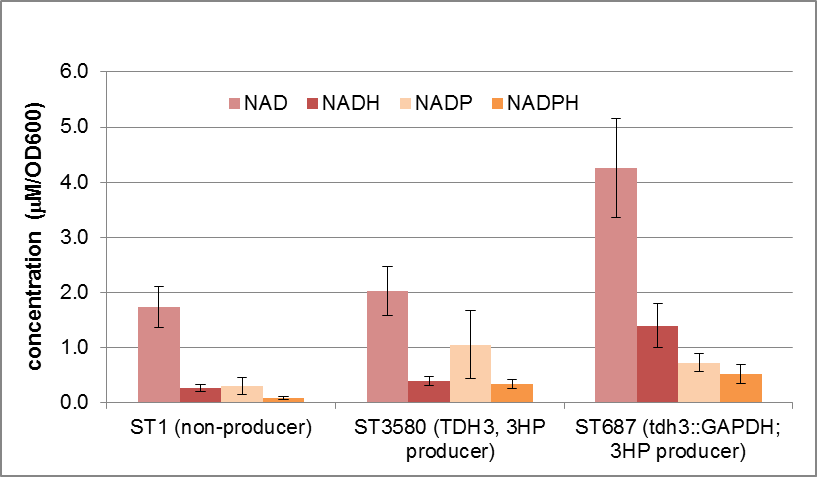
**

**Figure S3.** Intracellular concentrations of redox cofactors in the engineered strains. Data are mean and + standard deviations from biological triplicates.

**
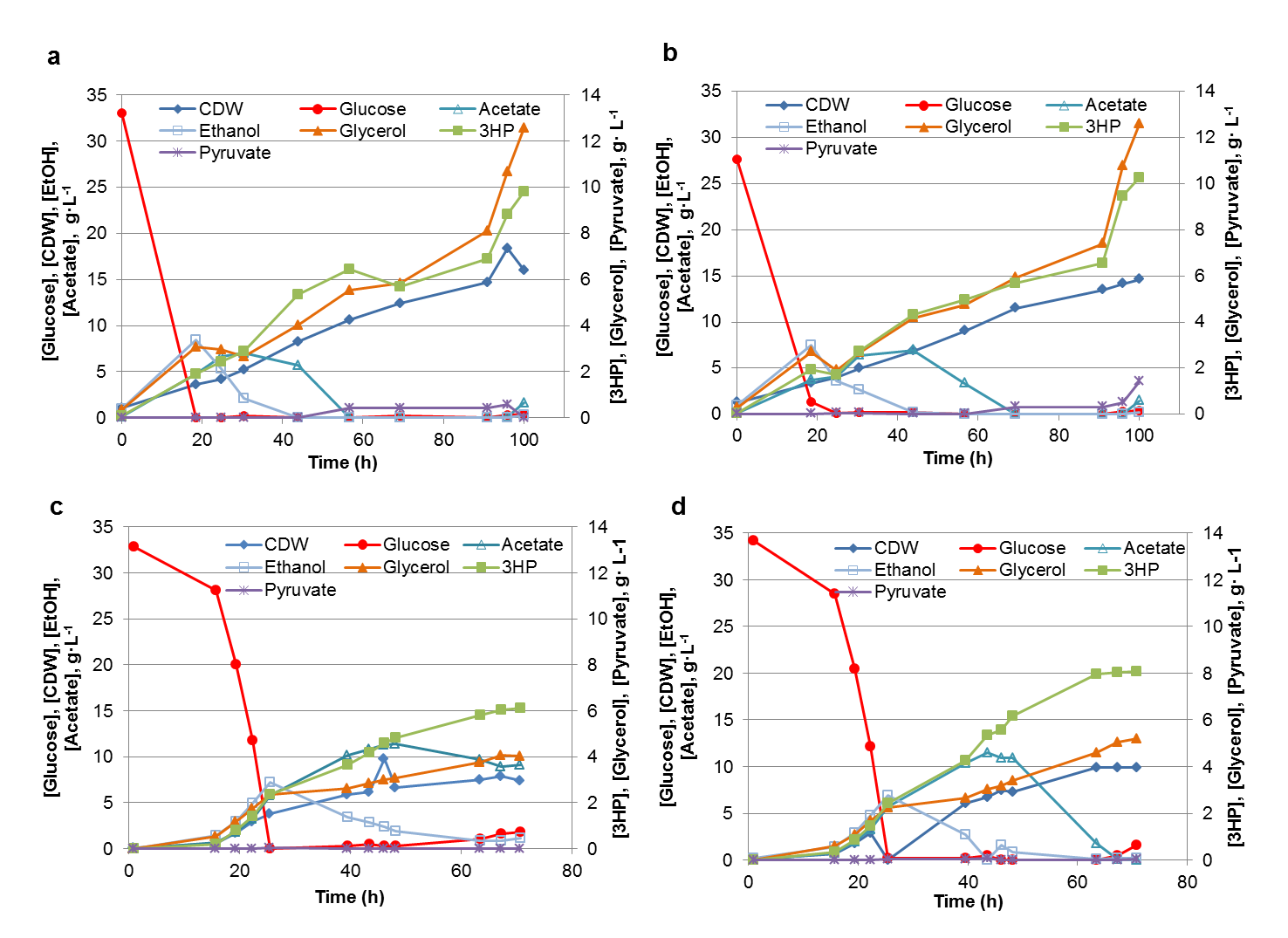
**

**Figure S4.** Growth and metabolites production profiles of the best 3HP-producing strain ST687 grown in C-limited fed-batch fermentation (**a** and **b**) and N/C-limited fed-batch fermentation (**c** and **d**). The cultivations were performed in triplicates; here the replicates of fermentations from Figure 4 are shown.

**Supplementary Tables**

**Table S1.** Oligonucleotide sequences. Underlined sequences represent overhangs used in USER cloning.

| **Primer name** | **Sequence (5’ ->3’)** |
| --- | --- |
| ID53_ACC1**_fw | CGTGCGAUTCATTTCAAAGTCTTCAACAATTT |
| ID54_ACC1**_rv | AGTGCAGGUAAAACAATGAGCGAAGAAAGCTTA |
| ID176_CaMCR_fw_NEW | ATCTGTCAUAAAACAATGAGTGGTACAGGTAG |
| ID177_CaMCR_rv_NEW | CACGCGAUTCAGACTGTAATGGCTCTACCTC |
| ID5_PTEF1_fw | ACCTGCACUTTGTAATTAAAACTTAG |
| ID6_PTEF1_rv | CACGCGAUGCACACACCATAGCTTC |
| ID7_PPGK1_fw | cgtgcgauggaagtaccttcaaaga |
| ID8_PPGK1_rv | ATGACAGAUTTGTTTTATATTTGTTG |
| ID644_ACSse_U1_fw | AGTGCAGGUAAAACAATGTCACAAACACAC |
| ID645_ACSse_U1_rv | CGTGCGAUTCATGATGGCATAGCAATAG |
| ID738_ALD6_U2_fw | ATCTGTCAUAAAACAATGACTAAGCTACACTTTGACAC |
| ID739_ALD6_U2_rv | CACGCGAUTCACAACTTAATTCTGACAGCTTTTAC |
| ID1187_PDC1_U1longer_fw | AGTGCAGGUAAAACAATGTCTGAAATTACTTTGGGtaaatatttg |
| ID1188_PDC1_U1longer_rv | CGTGCGAUTCATTGCTTAGCGTTGGTAGCAGCAGTC |
| ID092_URA3-DW-rev | CGCTTCCCATCCAGCATTTC |
| ID093_URA3-UP-rev | CTGTCGTTCCATTGAAAGC |
| ID141_marker-UP-fwd | AGAACAGCUGAAGCTTCGTACG |
| ID150_KlLEU2-UP-rev | CAGAAGCATAACTACCCATTCC |
| ID142_marker-DW-rev | AGGCCACUAGTGGATCTGATATCAC |
| ID151_KlLEU2-DW-fwd | TGGAAGAGGCAAGCACGTTAGC |
| ID504_TDH3-UP-FWD1 | GCAATTGACCCACGCATGTA |
| ID506_TDH3-UP-Rev | AGCTGTTCUCGAAACCGTTAATAGCAACTC |
| ID507_TDH3-DW-fwd | AGTGGCCUACGTTGCCAAGGCTTAAGTG |
| ID508_TDH3-DW-rev1 | GGAAGAAATGAGGATTGAGC |
| ID337_CaGAPDH-fwd | ACAAAACAAAAUGGCAAAGATAGCTATTAATGG |
| ID322_CaGAPDH-rev | AGCTGTTCUCTATTTTGCTATTTTTGCAAAGTAAGC |
| ID10657_tdh3UPrevU2 | ATTTTGTTTTGUATGTGTGTTTATTCGAAACTAAG |
| ID155_TDH1-UP-rev | ATTTTGTTTTGUGTGTAAATTTAGTGAAGTACTG |
| ID157_TDH1-UP-fwd2 | AGTTGGGCTGAGCTTCTGATCC |
| ID160_TDH1-DW-fwd | AGTGGCCUCTTGATCGAATATGTTGCCAAGGC |
| ID161_TDH1-DW-rev1 | AGAGCACTTCCGAACTTGATCC |
| ID163_TDH2-UP-rev | ATTTTGTTTTGUTTGTTTGTGTGATG |
| ID164_TDH2-UP-fwd1 | GTACCAACATCGGTTGAAACAG |
| ID166_TDH2-DW-fwd | GGAGTTAAAUTTAAGCCTTGGCAACGAGGCCACT |
| ID167_TDH2-DW-Rev1 | CATTCAAGAGGAAACAGAAGTGG |
|  |  |

**Table S1. (Continue)**

| **Primer name** | **Sequence (5’ ->3’)** |
| --- | --- |
| **Genotying primers** |  |
| ID903_X-3-up-out | TGACGAATCGTTAGGCACAG |
| ID905_X-4-up-out | CTCACAAAGGGACGAATCCT |
| ID2221_ColoPCR_vec_TADH1_towards out | GTTGACACTTCTAAATAAGCGAATTTC |
| ID505_TDH3-UP-test-fwd | CCTCTTAACAGGTTCAGACG |
| ID509_TDH3-DW-rev2 | GACTTCTCTGTTCCCATTAGG |
| ID156_TDH1-UP-fwd1 | GTAACCACACCACATTTTCAGG |
| ID162_TDH1-DW-rev2 | TGGTCGTGGAACTTGCATAGC |
| ID165_TDH2-UP-fwd2 | TATCTTGACGGGTATTCTGAGC |
| ID168_TDH2-DW-Rev2 | CATTCCTTAGAGATGCAGCTCC |
| **qPCR primers** |  |
| ID434_ALG9_fw | CACGGATAGTGGCTTTGGTGAACAATTAC |
| ID435_ALG9_rv | TATGATTATCTGGCAGCAGGAAAGAACTTGGG |
| ID980_ACC1**_fw | TTGTTAAGAGTCACGGTGGT |
| ID981_ACC1**_rv | CGGCCATACGGATATATTCTG |
| ID982_CaMCR_fw | TGCCTTGGCTGAAAGAATG |
| ID983_CaMCR_rv | CCTGGACCCAATTCTGCTT |

**Table S2.** Oligos and templates used to generate gene fragments for USER cloning and yeast transformation by PCR

| Fragment ID | Description | Oligo forward | Oligo Reverse | Template |
| --- | --- | --- | --- | --- |
| BB12_ACC1**<- | *ACC1^S659A, S1157A^* from *S. cerevisiae* (*ACC1^**^*) | ID53 | ID54 | pSP-GM2-ACC1^S659A, S1157A^ |
| BB11_->CaMCR | Malonyl-CoA reductase from *Chloroflexus aurantiacus* | ID176 | ID177 | pYC6 |
| BB10_<-P_TEF1_-P_PGK1_-> | Fused promoters of *TEF1* and *PGK1* genes from *S. cerevisiae* | ID5 | ID8 | plasmid pSP-GM1 |
| BB157_ACC1**<-pTEF-pPGK->CaMCR | *ACC1***<-P_TEF1_-P_PGK1_->*CaMCR* | ID53 | ID177 | pCfB298 |
| BB119_*SEacs^L641P^* <- | Acetyl-CoA synthetase from *Salmonella enterica (SEacs^L641P^)* | ID644 | ID645 | pCfB324 |
| BB158_ALD6-> | Acetaldehyde dehydrogenase 6 from *S. cerevisiae* | ID738 | ID739 | *S. cerevisiae* gDNA (CEN.PK102-5B) |
| BB159_PDC1<- | Pyruvate decarboxylase isozyme 1 from *S. cerevisiae* | ID1187 | ID1188 | *S. cerevisiae* gDNA (CEN.PK102-5B) |
| BB8_<-P_TEF1_ | *TEF1* promoter from *S. cerevisiae* | ID5 | ID6 | *S. cerevisiae* gDNA (CEN.PK102-5B) |
| BB43_KlURA3-UP | Upper part of LoxP-KlURA3-LoxP selection marker cassette | ID141 | ID093 | pUG72 |
| BB44_KlURA3-DW | Down part of LoxP-KlURA3-LoxP selection marker cassette | ID092 | ID142 | pUG72 |
| BB47_KlLEU2-UP | Upper part of LoxP-KlLEU2-LoxP selection marker cassette | ID141 | ID150 | pUG73 |
| BB48_KlLEU2-DW | Down part of LoxP-KlLEU2-LoxP selection marker cassette | ID151 | ID142 | pUG73 |
| tdh3-null-UP | Upstream part of *TDH3* locus | ID504 | ID506 | *S. cerevisiae* gDNA (CEN.PK102-5B) |
| tdh3-DW | Downstream part of *TDH3* locus | ID507 | ID508 | *S. cerevisiae* gDNA (CEN.PK102-5B) |
| tdh3-null-up-LEU2 | UP transformation fragment for deletion of TDH3 | ID504 | ID150 | tdh3-null-up + BB47 |
| tdh3-DW-LEU2 | DW transformation fragment for deletion/CDS exchange of TDH3 | ID151 | ID508 | BB48 + tdh3-DW |
| BB93-CaGAPDH | *CaGAPDH* for *TDH::CaGAPDH* | ID337 | ID322 | *Clostridium acetobutylicum* gDNA |
| tdh3-cdsex-UP | Upstream part of TDH3 for CDS exchange | ID504 | ID10657 | *S. cerevisiae* gDNA (CEN.PK102-5B) |
| tdh3::CaGAPDH-UP-LEU | UP transformation fragment for tdh3::CaGAPDH | ID504 | ID150 | tdh3-cdsex-UP + BB93 + BB47 |
| BB52-tdh2-UP | Upstream part of TDH2 for CDS exchange | ID164 | ID163 | *S. cerevisiae* gDNA (CEN.PK102-5B) |
| BB53-tdh2-DW | Downstream part of TDH2 locus | ID166 | ID167 | *S. cerevisiae* gDNA (CEN.PK102-5B) |
| tdh2::CaGAPDH-UP-LEU | UP transformation fragment for tdh2::CaGAPDH | ID164 | ID150 | BB52-tdh2-UP + BB93 + BB47 |
| tdh2-DW-LEU | DW transformation fragment for tdh2::CaGAPDH | ID151 | ID167 | BB53-tdh2-DW + BB48 |
| BB49-tdh1-UP | Upstream part of TDH2 for CDS exchange | ID157 | ID155 | *S. cerevisiae* gDNA (CEN.PK102-5B) |
| BB50-tdh1-DW | Downstream part of TDH2 locus | ID160 | ID161 | *S. cerevisiae* gDNA (CEN.PK102-5B) |
| tdh1::CaGAPDH-UP-URA | UP transformation fragment for tdh1::CaGAPDH | ID157 | ID093 | BB49-tdh1-UP + BB93 + BB43 |
| tdh1-DW-URA | DW transformation fragment for tdh1::CaGAPDH | ID092 | ID161 | BB50-tdh1-DW + BB44 |

**Table S3. Plasmids**

| **Plasmid name** | **Parent plasmid** | **Description** | **Reference/Source** |
| --- | --- | --- | --- |
| pCfB54 | - | pESC-URA-USER | Jensen *et al.*, 2014 [1] |
| pCfB255 | - | pX-2-LoxP-KlURA3 | Jensen *et al.*, 2014 [1] |
| pCfB257 | - | pX-3-LoxP-KlLEU2 | Jensen *et al.*, 2014 [1] |
| pCfB258 | - | pX-4-LoxP-SpHIS5 | Jensen *et al.*, 2014 [1] |
| pCfB322 | - | pTY4-LoxP-KlURA3tag | Borodina *et al*., 2014 [2] |
| pSP-GM1 | - | plasmid contains double promoters P_TEF1_-P_PGK1_ | Chen *et al*., 2014 [3] |
| pSP-GM2- ACC1^Ser659Ala, Ser1157Ala^ | - | Plasmid contains *ACC1^**^* from *S. cerevisiae* | Shi *et al.,* 2014 [4] |
| pYC1 | - | Plasmid contains *MCR* from *Chloroflexus aurantiacus* (*CaMCR*) | Chen *et al*., 2014 [3] |
| pYC9 | - | Plasmid contains *ACS* from *Salmonella enterica* (*SEacs^L641P^*) | Chen *et al*., 2014 [3] |
| pUG73 | - | Plasmid contains LoxP-KlLEU2 | Euroscarft |
| pCfB298 | pCfB54 | pESC-URA-ACC1**<-P_TEF1_-P_PGK1_-> CaMCR | This study |
| pCfB343 | pCfB255 | pX-2-LoxP-KlURA3tag- ACC1**<-P_TEF1_-P_PGK1_-> CaMCR | This study |
| pCfB376 | pCfB322 | pTY4-LoxP-KlURA3- ACC1**<-P_TEF1_-P_PGK1_-> CaMCR | This study |
| pCfB474 | pCfB376 | pTY4- KlURA3- ACC1**<-P_TEF1_-P_PGK1_-> CaMCR (removed LoxP sites from pCfB376) | This study |
| pCfB380 | pCfB257 | pX-3-LoxP-KlLEU2 - *SEacs^L641P^* <-P_TEF1_-P_PGK1_-> ALD6 | This study |
| pCfB382 | pCfB258 | pX-4-LoxP-SpHiS5-PDC1<-P_TEF1_ | This study |

**Table S4. Yeast strains**

| **Strain** | **Parent strain** | **Insertion fragments/plasmid** | **Genotype** | **Reference/ Source** |
| --- | --- | --- | --- | --- |
| CEN.PK113-7D (ST1; reference strain) | **-** | **-** | MATa *URA3-52 HIS3 LEU2 TRP1 MAL2-8^c^ SUC2* | Peter Kötter |
| CEN.PK102-5B | - | - | MATa *ura3-52 his3 Δ1 leu2-3/112* MAL2-8c SUC2 [ura^-^ his^-^ leu^-^] | Peter Kötter |
| CEN.PK102-5D | - | - | MATa *ura3-52 HIS3 LEU2 TRP1 MAL2-8^c^ SUC2* [ura^-^] | Peter Kötter |
| tdh3-null | CEN.PK102-5B | tdh3-null-up-LEU2 +  tdh3-DW-LEU2 | MATa ura^-^ his^-^ leu^-^ tdh3::LoxP | This study |
| tdh1::CaGAPDH, tdh3-null | tdh3-null | tdh1::CaGAPDH-UP-URA + tdh1-DW-URA | MATa ura^-^ his^-^ leu^-^ *tdh1::CaGAPDH-LoxP,tdh3::LoxP* | This study |
| tdh2::CaGAPDH, tdh3-null | tdh3-null | tdh2::CaGAPDH-UP-LEU + tdh2-DW-LEU | MATa ura^-^ his^-^ leu^-^ *tdh2::CaGAPDH-LoxP,tdh3::LoxP* | This study |
| tdh1+2::CaGAPDH, tdh3-null | tdh2::CaGAPDH, tdh3-null | tdh1::CaGAPDH-UP-URA + tdh1-DW-URA | MATa ura^-^ his^-^ leu^-^ *tdh1::CaGAPDH-LoxP,tdh2::CaGAPDH-LoxP,tdh3::LoxP* | This study |
| tdh3::CaGAPDH | CEN.PK102-5B | tdh3::CaGAPDH-UP-LEU + tdh3-DW-LEU | MATa ura^-^ his^-^ leu^-^ *tdh3::CaGAPDH-LoxP* | This study |
| tdh1+3::CaGAPDH | tdh3::CaGAPDH | tdh1::CaGAPDH-UP-URA +tdh1-DW-URA | MATa ura^-^ his^-^ leu^-^ *tdh1::CaGAPDH-LoxP,tdh3::CaGAPDH-LoxP* | This study |
| tdh2+3::CaGAPDH | tdh3::CaGAPDH | tdh2::CaGAPDH-UP-LEU +tdh2-DW-LEU | MATa ura^-^ his^-^ leu^-^ *tdh2::CaGAPDH-LoxP,tdh3::CaGAPDH-LoxP* | This study |
| tdh1+2+3::CaGAPDH | tdh2+3::CaGAPDH | tdh1::CaGAPDH-UP-URA +tdh1-DW-URA | MATa ura^-^ his^-^ leu^-^ *tdh1::CaGAPDH-LoxP,tdh2::CaGAPDH-LoxP,tdh3::CaGAPDH-LoxP* | This study |
| 3HP-M1 | CEN.PK102-5D | pCfB298 | MATa *ura3-52,* 2 μ, *P_TEF1_::ACC1** P_PGK1_::CaMCR URA3* | This study |
| 3HP-M2 | CEN.PK102-5D | pCfB343 | MATa *ura3-52, P_TEF1_::ACC1** P_PGK1_::CaMCR KlURA3* | This study |
| 3HP-M3 | CEN.PK102-5D | pCfB474 | MATa *ura3-52, (P_TEF1_::ACC1** P_PGK1_::CaMCR KlURA3tag)n* | This study |
| 3HP-M4 | CEN.PK102-5B | pCfB474 pCfB257 pCfB258 | MATa ura^-^ his^-^ leu^-^ *(P_TEF1_::ACC1** P_PGK1_::CaMCR KlURA3tag)n* *LoxP-KlLEU2, LoxP-SpHIS5* | This study |
| 3HP-M5 | CEN.PK102-5B | pCfB380 pCfB474 pCfB258 | MATa ura^-^ his^-^ leu^-^ *P_TEF1_:: SEacs^L641P^ P_PGK1_::ALD6 KlLEU2*  *(P_TEF1_::ACC1** P_PGK1_::CaMCR KlURA3tag)n, LoxP-SpHIS5* | This study |
| 3HP-M6 | CEN.PK102-5B | pCfB382  pCfB380 pCfB474 | MATa ura^-^ his^-^ leu^-^ *P_TEF1_:: PDC1 SpHIS5*  *P_TEF1_:: SEacs^L641P^ P_PGK1_::ALD6 KlLEU2*  *(P_TEF1_::ACC1** P_PGK1_::CaMCR KlURA3tag)n* | This study |
| 3HP-M7 | tdh3-null | pCfB382  pCfB380 pCfB474 | MATa ura^-^ his^-^ leu^-^ tdh3 ∆ *P_TEF1_:: PDC1 SpHIS5*  *P_TEF1_:: SEacs^L641P^ P_PGK1_::ALD6 KlLEU2*  *(P_TEF1_::ACC1** P_PGK1_::CaMCR KlURA3tag)n* | This study |
| 3HP-M8 | tdh3-null tdh1::CaGAPDH | pCfB382  pCfB380 pCfB474 | MATa ura^-^ his^-^ leu^-^ tdh3 ∆ tdh1::CaGAPDH, *P_TEF1_:: PDC1 SpHIS5*  *P_TEF1_:: SEacs^L641P^ P_PGK1_::ALD6 KlLEU2*  *(P_TEF1_::ACC1** P_PGK1_::CaMCR KlURA3tag)n* | This study |
| 3HP-M9 | tdh3-null tdh2::CaGAPDH, | pCfB382  pCfB380 pCfB474 | MATa ura^-^ his^-^ leu^-^ tdh3 ∆ tdh2::CaGAPDH, *P_TEF1_:: PDC1 SpHIS5*  *P_TEF1_:: SEacs^L641P^ P_PGK1_::ALD6 KlLEU2*  *(P_TEF1_::ACC1** P_PGK1_::CaMCR KlURA3tag)n* | This study |
| 3HP-M10 | tdh3-null tdh1+2::CaGAPDH | pCfB382  pCfB380 pCfB474 | MATa ura^-^ his^-^ leu^-^ tdh3 ∆ *tdh1+2::CaGAPDH, P_TEF1_:: PDC1 SpHIS5*  *P_TEF1_:: SEacs^L641P^ P_PGK1_::ALD6 KlLEU2*  *(P_TEF1_::ACC1** P_PGK1_::CaMCR KlURA3tag)n* | This study |
| 3HP-M11 | tdh3::CaGAPDH | pCfB382  pCfB380 pCfB474 | MATa ura^-^ his^-^ leu^-^  *tdh3::CaGAPDH P_TEF1_:: PDC1 SpHIS5*  *P_TEF1_:: SEacs^L641P^ P_PGK1_::ALD6 KlLEU2*  *(P_TEF1_::ACC1** P_PGK1_::CaMCR KlURA3tag)n* | This study |
| 3HP-M12 | tdh1+3::CaGAPDH | pCfB382  pCfB380 pCfB474 | MATa ura^-^ his^-^ leu^-^  *tdh1+3::CaGAPDH P_TEF1_:: PDC1 SpHIS5*  *P_TEF1_:: SEacs^L641P^ P_PGK1_::ALD6 KlLEU2*  *(P_TEF1_::ACC1** P_PGK1_::CaMCR KlURA3tag)n* | This study |
| 3HP-M13 | tdh2+3::CaGAPDH | pCfB382  pCfB380 pCfB474 | MATa ura^-^ his^-^ leu^-^  *tdh2+3::CaGAPDH P_TEF1_:: PDC1 SpHIS5*  *P_TEF1_:: SEacs^L641P^ P_PGK1_::ALD6 KlLEU2*  *(P_TEF1_::ACC1** P_PGK1_::CaMCR KlURA3tag)n* | This study |
| 3HP-M14 | tdh1+2+3::CaGAPDH | pCfB382  pCfB380 pCfB474 | MATa ura^-^ his^-^ leu^-^  *tdh1+2+3::CaGAPDH  P_TEF1_:: PDC1 SpHIS5*  *P_TEF1_:: SEacs^L641P^ P_PGK1_::ALD6 KlLEU2*  *(P_TEF1_::ACC1** P_PGK1_::CaMCR KlURA3tag)n* | This study |

**Table S5.** MRM transitions for NAD^+^, NADH, NADP^+^ and NADPH

|  |  | Precursor ion | Product ion | CE, eV | Product ion | CE, eV |
| --- | --- | --- | --- | --- | --- | --- |
| NAD | [ESI]+ | 664 | 428 | 23 | 136 | 33 |
| NADH | [ESI]+ | 666 | 643 | 15 | 514 | 25 |
| NADP | [ESI]+ | 744 | 604 | 20 | 622 | 12 |
| NADPH | [ESI]+ | 745 | 605 | 17 | 623 | 12 |

**References**

1. Jensen NB, Strucko T, Kildegaard KR, David F, Maury J, Mortensen UH, Forster J, Nielsen J, Borodina I. EasyClone: method for iterative chromosomal integration of multiple genes in *Saccharomyces cerevisiae*. FEMS Yeast Res. 2014,14:238–248.

2. Borodina I, Kildegaard KR, Jensen NB, Blicher TH, Maury J, Sherstyk S, Schneider K, Lamosa P, Herrgård MJ, Rosenstand I, Öberg F, Forster J, Nielsen J. Establishing a synthetic pathway for high-level production of 3-hydroxypropionic acid in *Saccharomyces cerevisiae* via β-alanine. Metab Eng. 2015,27:57–64.

3. Chen Y, Bao J, Kim I-K, Siewers V, Nielsen J. Coupled incremental precursor and co-factor supply improves 3-hydroxypropionic acid production in *Saccharomyces cerevisiae*. Metab Eng. 2014,22:104–109.

4. Shi S, Chen Y, Siewers V, Nielsen J. Improving Production of Malonyl Coenzyme A-Derived Metabolites by Abolishing Snf1-Dependent Regulation of Acc1. mBio. 2014,5:e01130–14.

1. * These authors contributed equally to the work.

   Present address: Evolva Biotech A/S, Lersø Park Allé 42-44, DK-2100 Copenhagen Ø, Denmark.

   2 Present address: Bio R&D Center, Paikkwang Industrial Co. Ltd., 57 Oehang-4 gil, Gunsans-si, Jellabukdo,
    Korea.

   # Corresponding author. Fax: +45 4525 80 01. *E-mail address*: irbo@biosustain.dtu.dk. [↑](#footnote-ref-1)
2. [↑](#footnote-ref-2)
